# A family with Milroy disease caused by the *FLT4/VEGFR3* gene variant c.2774 T > A

**DOI:** 10.1186/s12920-021-00997-w

**Published:** 2021-06-08

**Authors:** Yu Sui, Yongping Lu, Meina Lin, Xiang Ni, Xinren Chen, Huan Li, Miao Jiang

**Affiliations:** Key Laboratory of Reproductive Health and Medical Genetics, Liaoning Province Research Institute of Family Planning, China Medical University, 10 Puhe Street, Huanggu District, Shenyang, 110031 Liao Ning Province China

**Keywords:** Milroy disease, *FLT4/VEGFR3*, Heterogeneity

## Abstract

**Background:**

Milroy disease (MD) is a rare, autosomal-dominant disorder. Variants in the Fms-related tyrosine kinase 4 (*FLT4/VEGFR3*) gene cause the symptoms of this disease. In this report, we investigated the variant in a large Chinese family with MD.

**Methods:**

We conducted Sanger sequencing of exons 17–26 of *FLT4/VEGFR3* (NM_182925.4). We assessed its pathogenicity based on the ACMG criteria and predicted it with an in silico program.

**Results:**

A heterozygous substitution (NM_182925.4 (*FLT4/VEGFR3*):c.2774 T>A, p. (Val925Glu)) was detected in all patients with MD but not in any healthy controls. The variant was evaluated as pathogenic according to the ACMG criteria and was predicted to be pathogenic using an in silico program.

**Conclusions:**

In this report, we described a large family with MD caused by a missense variant in *FLT4/VEGFR3* (NM_182925.4 (*FLT4/VEGFR3*_v001):c.2774 T>A, p. (Val925Glu)). There are phenotypic heterogeneities among family members, and further research should be conducted to explore the possible reasons.

**Supplementary Information:**

The online version contains supplementary material available at 10.1186/s12920-021-00997-w.

## Background

Primary hereditary lymphoedema type IA (LMPH1A, OMIM#153100), also known as Milroy disease (MD), is a rare, autosomal-dominant disorder [1]. Characteristic symptoms include painless and nonprogressive lymphoedema with uni- or bilateral oedema of the legs and feet [2–4] and prominent venous vessels [2], congenital hand oedema [5], persistent bilateral pleural effusion with high protein levels [2–4] and scrotal swelling [2]. Fms-related tyrosine kinase 4 (*FLT4)* (also known as vascular endothelial growth factor receptor 3, *VEGFR3*) encodes a receptor tyrosine kinase [6–9] and is important for lymphatic endothelial cell survival, proliferation and migration [10, 11]. Variants of *FLT4/VEGFR3* impair tyrosine kinase signalling and cause MD [12]. In this study, we explored a large Chinese family with MD to identify the pathogenic variant.

## Methods

### Pedigree construction

The propositus with suspected primary lymphoedema was self-referred to our research team and was examined by lymphological specialists and medical geneticists (Miao Jiang). Then, we constructed a family history. All participants underwent a series of clinical examinations for primary lymphoedema. The recruited patients satisfied at least one of the following criteria: lower extremity lymphoedema, pitting oedema, hyperkeratosis and subcutaneous thickening. We also assessed the nail plate morphology of the patients. Secondary lymphoedema was excluded after medical history inquiries; The patients were asked if they had filariasis, cancer, infection, radio-/chemotherapy and surgery. Genetic counselling was offered to the family members when the pathogenic variant was identified. The Ethnic Committee of the Research Institute of Family Planning approved the study protocol.

### DNA extraction

Blood samples were obtained from all family members. We recruited 100 healthy individuals from the same geographical areas as the patients to clarify whether the possible variant was an innocuous polymorphism or pathogenic variant. Genomic DNA was extracted from the blood samples using a DNA Isolation Kit for Mammalian Blood (Tiangen Biotech, China).

### PCR amplification and Sanger sequencing

We sequenced exons 17–26 (tyrosine kinase coding domains) of *FLT4/VEGFR3* in all family members (Sangon Biotech, Shanghai, China). The primers and conditions for the PCR amplification of *FLT4/VEGFR3* (NM_182925.4, http://www.ncbi.nlm.nih.gov/Refseq/) are provided in the supplementary materials (Additional file 4: Table S1). The primers for the amplification of exon 20 were as follows: forward primer, 5′ CTTCATCAGCGTCGAGTGG 3′ and reverse primer, 5′ ATTATGGGCGGGTTCCTT 3′. The PCR conditions for the amplification of exon 20 of *FLT4/VEGFR3* were as follows: denaturing at 95 °C for 5 min; 35 cycles of denaturing at 95 °C for 30 s, annealing at 58 °C for 30 s, and extension at 72 °C for 30 s; and a final step for 7 min at 72 °C. The amplified fragment was 176 bp. The amplification system was as follows: 2 × Biotech Power PCR Mix, 10 µl; forward primer, 0.8 µl (10 µM); reverse primer, 0.8 µl (10 µM); DNA template, 1 µl (50 ng/µl); and ddH2O, 12.4 µl. The amplification reaction was 25 µl. The novel variant was also ruled out as a polymorphism by digestion with the restriction enzyme HphI, and the amplification primers were as follows: 5′ AACCTCCTCGGGGCGTGCACCAAGC 3′ and 5′ GCGCAGGGGCTGAAGGCGTCCCG 3′. The amplification system was as follows: 2 × Biotech Power PCR Mix, 10 µl; forward primer, 0.8 µl (10 µM); reverse primer, 0.8 µl (10 µM); DNA template, 1 µl (50 ng/µl); and ddH2O, 12.4 µl. The PCR amplification conditions for *FLT4/VEGFR3* were denaturing at 98 °C for 30 s; 35 cycles of denaturing at 98 °C for 30 s, annealing at 58 °C for 30 s, extension at 72 °C for 30 s; and a final step for 7 min at 72 °C. The PCR amplification fragment was 262 bp.

### Variant analysis

We evaluated the pathogenicity of the variant based on the ACMG criteria and an in silico program (MutationTaster, MutationAssessor, PolyPhen HDIV & HVAR, FATHMM, PROVEAN, M-CAP, and SIFT [13–19]). We searched human FLT4/VEGFR3 protein homologues using BLAST on the NCBI website (http://www.ncbi.nlm.nih.gov). The identified proteins were aligned using ClustalW [20], and a phylogenetic tree was reconstructed with MEGA4 with the neighbour-joining method [21].

### Informed consent of the research

All participants enrolled in this study provided written consent to participate after being informed of the nature of the research. All family members enrolled in the study signed two informed consent forms: one for the genetic test and the other to make the clinical and genetic data available for research purposes. Signed informed consent was obtained from all members of the studied family for the publication of personal and clinical information (images included) in this research.

## Results

### Description of the MD pedigree

**Fig. 1:**
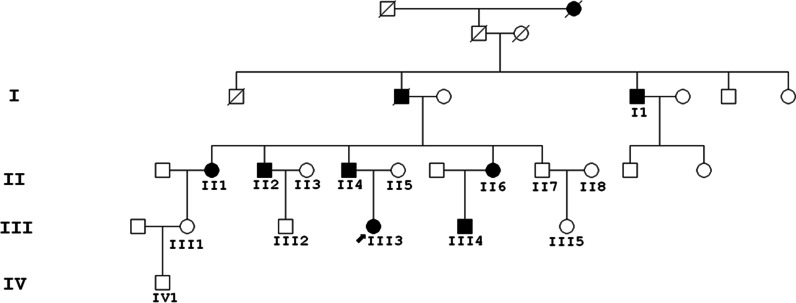
Pedigree of the Milroy disease family. Black symbols represent the affected individuals, arrow indicates the proband

**Fig. 2:**
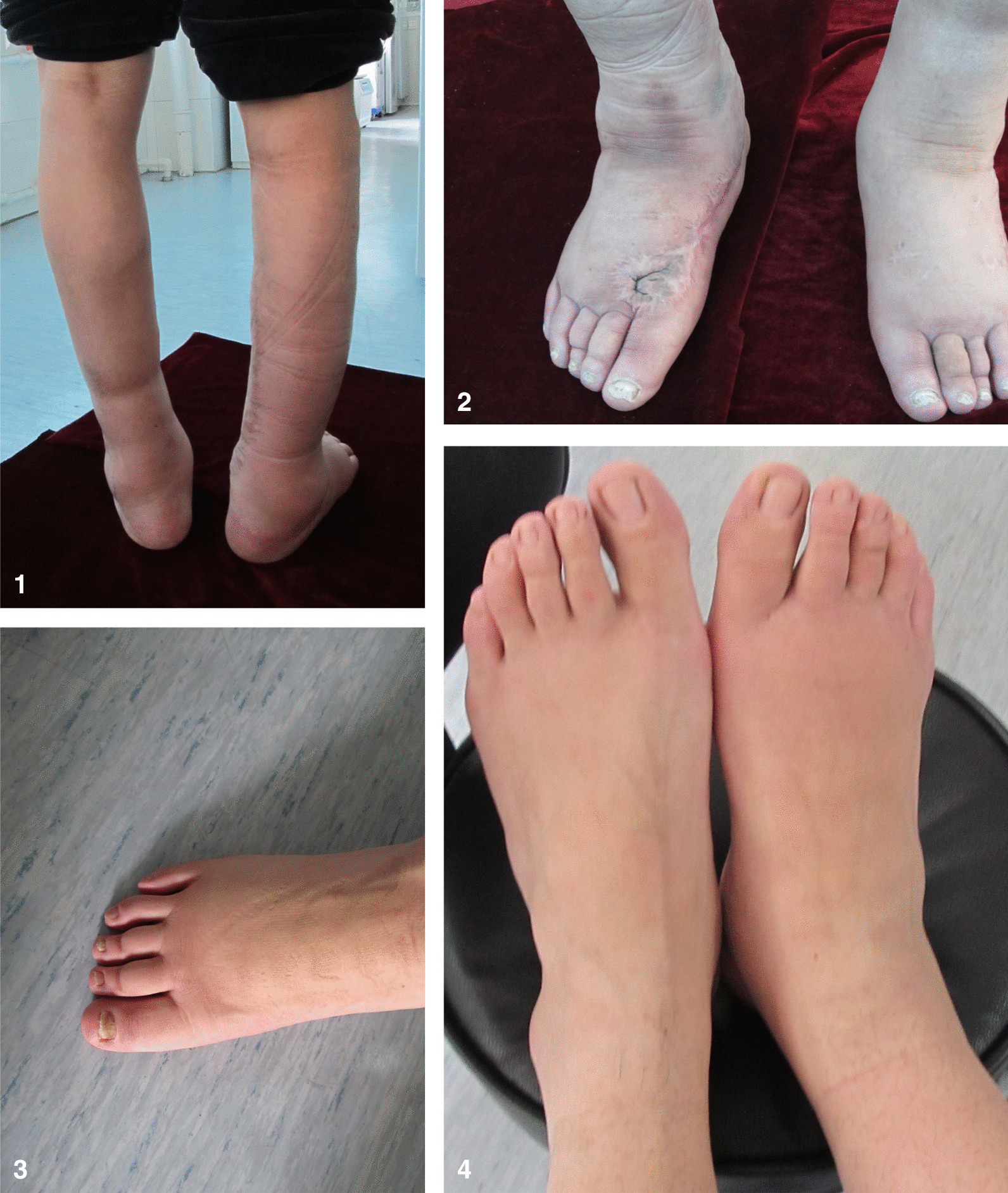
Bilateral lymphedema of lower limbs of Milroy disease family. **1**, **2** Bilateral lymphedema of lower limbs of Patient II1; **3** bilateral lymphedema of lower limbs of Patient II6; **4** bilateral lymphedema of lower limbs of Patient III3

**Fig. 3:**
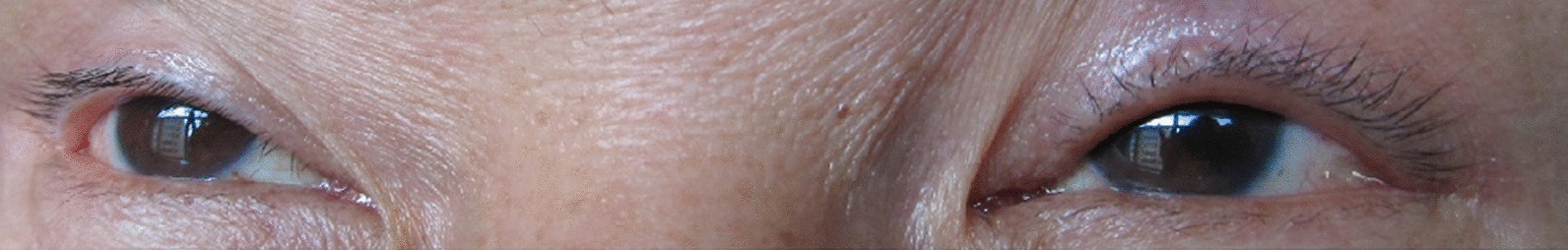
Slight epicanthic folds and down-slanting palpebral fissures of Patient II4

**Fig. 4:**
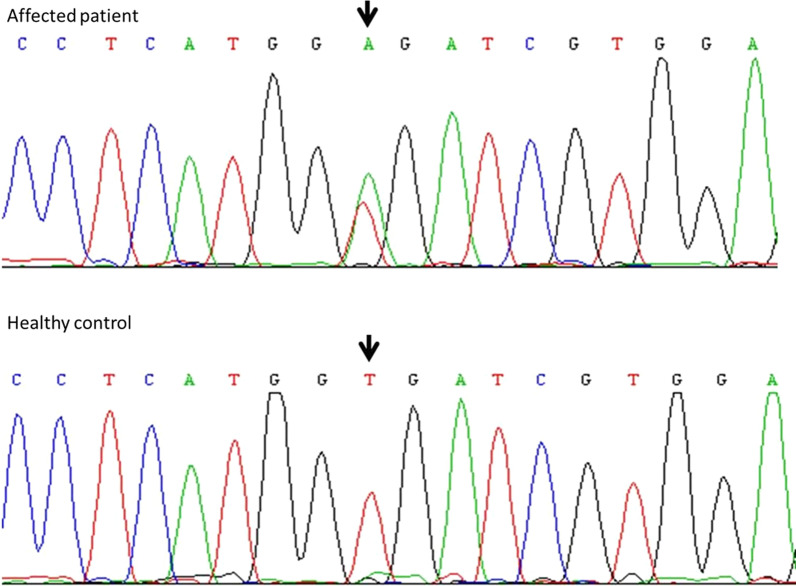
Sanger sequencing showing a heterozygous change c.2774 C>T of FLT4 gene. Sanger sequence analysis of an affected individual and a normal unaffected control. The mutation shown by black arrow

The pedigree had 4 generations of Chinese patients, all living in Southeast China (Shenyang City, Liaoning Province), and the pedigree included 7 patients (age ranging from 29 to 75 years old) and 8 healthy controls (Fig. 1). Table 1 shows the clinical features of all 7 patients (Table 1). All the affected individuals presented with congenital bilateral lower limb lymphoedema at birth, and the lymphoedema extended from the toes to the upper calves, presenting as different degrees of creases and a brawny texture of the skin (Fig. 2-1, 2, 3, 4). The propositus (III3) always felt pain in her foot after taking a long walk, especially during the hot season. The swollen region was also warm to the touch, and marks caused by compression stockings were visible (Fig. 2-1, 2, 3, 4). However, the phenotype of her male cousin (III4) could not be observed by the naked eye. In contrast, patient I1 complained that the oedema of his legs was aggravated and extended to the roots of the thighs when he had a cold and fever in childhood. Patient II1 complained that during childhood, skin hyperkeratosis of the feet occurred, and lymphoedema caused the instep to rise too high to wear shoes. The subcutaneous tissue of the foot was filled with lymphatic fluid, causing local foot and tissue swelling and the deformation and proliferation of adipose tissue and connective tissue (Fig. 2-1, 2). In adulthood, oedema continued to the lower extremities beneath the knees (Fig. 2-1, 2). Lymphoedema was not currently visible due to plastic surgery, but the heavy oedema of the lower extremities and elephant-like appearance of her legs were obvious (Fig. 2-1, 2). No other patients (II1, II2, II4, II6, III3, and III4) showed similar phenotypes under the same conditions in this family; they showed lower levels of keratinization of the foot skin and lighter swelling of the lower limbs. Hypoplastic toenails with upturned concavity were present among the patients and were consistent with “ski jump” nails (Fig. 2-3: Bilateral lower limb lymphoedema of patient II6). The father of propositus (II4) showed slight epicanthic folds and downslanting palpebral fissures (Fig. 3) [4]. No asymptomatic carriers, late onset or nonpenetrant cases were found in the family. No hydrocoele was found in the male members.

**Table 1:** Overview of clinical features of 7 patients

Patient	Onset at birth	Congenital bilateral lower limbs lymphedema at birth	Deep creases over the toes	Congenital hands edema	Brawny texture of the skin	Small dysplastic toenails ('ski jump')	Swelling of the scrotum	Hypoproteinemia	Lymphedematous	Edema extends to the thigh	Epicanthic folds and down-slanting palpebral fissures	Aggravated edema caused by cold	Tissue overgrowth, of connective and adipose tissue
I1	+	+	++	−	+	+	−	−	−	+	−	+	−
II1	+	+	++	−	+	+	−	−	−	−	−	−	+
II2	+	+	+	−	+	+	−	−	−	−	−	−	−
II4	+	+/−	+/−	−	+/−	+	−	−	−	−	+	−	−
II6	+	+	+	−	+	+	−	−	−	−	−	−	−
III3	+	+	+	−	+	+	−	−	−	−	−	−	−
III4	+	+/−	+/−	−	+/−	+	−	−	−	−	−	−	−

### Variant analyses

#### Identification of a novel variant in *FLT4/VEGFR3*

The Sanger sequencing results of exons 17–26 of *FLT4/VEGFR3* revealed a single nucleotide heterozygous substitution of T to A in all patients at nucleotide position 2774 in exon 20 that results in an amino acid change from valine to glutamic acid at amino acid residue 925 (NM_182925.4 (*FLT4/VEGFR3*_v001):c.2774 T>A, p.(Val925Glu)) (Fig. 4). The variant was detected in all affected patients (I1, II1, II2, II4, II6, III3 and III4) but not in unaffected family members (II3, II5, II7 II8, III1, III2, III5 and IV1). This variant was also ruled out as a polymorphism by HphI digestion, and the PCR fragment was 262 bp. The restriction endonuclease cleavage site is 5′GGTGA(N)_8_3′. If the variant site was present, the fragment was cleaved into two fragments, which were 198 bp and 64 bp, and analysed by 8% polyacrylamide gel electrophoresis and silver staining. We used DL2000 as the marker (TaKaRa). The polypropylene gel electrophoresis voltage was 400 V, and the electrophoresis time was 4 h. No variants were found in the 100 healthy controls or the healthy members of the family (Additional file 1: Figure S1). It was neither reported in global scale databases for variant annotation such as the Exome Aggregation Consortium (ExAC) nor in the 1000 Genomes Project. This novel variant was evaluated as “pathogenic” according to the ACMG criteria (2 moderate PMs: PM1 and PM2; 4 supporting PPs: PP1-4) and was predicted to be pathogenic using an in silico program (Additional file 5: Table S2). Twelve proteins in the FLT4/VEGFR3 subfamily were found by a BLAST search in the NCBI database (https://blast.ncbi.nlm.nih.gov/Blast.cgi) and formed a cluster in the phylogenetic tree (Additional file 2: Figure S2). A FLT4/VEGFR3 protein sequence alignment revealed the invariant valine in the tyrosine kinase domain, and the wild type is conserved in a wide range of organisms, ranging from humans to *Danio rerio* (Additional file 3: Figure S3).

## Discussion

Milroy disease (MD, OMIM: #153,100) is caused by developmental lymphatic vascular anomalies, with an estimated prevalence of 1 in 160,000 individuals [22]. MD patients usually exhibit lymphoedema at birth with the swelling of the lower limbs; most cases are bilateral [2] and, in some cases, swelling extends to the thighs [2, 23]. Patients often have a brawny texture and hyperkeratosis of the foot skin (Additional file 6: Table S3). Other phenotypes associated with MD included hydrocoele in males (37%), “ski jump” toenails (14%), and bilateral pleural effusion [2] (Additional file 6: Table S3).

*FLT4/VEGFR3* encodes a class III receptor tyrosine kinase, and it is predominantly expressed in lymphatic endothelial cells in adults and has dominant effects on lymphatic vessel growth. [6, 7, 24–30]. To date, nearly 196 variants have been reported, and most of them are missense variants (http://www.hgmd.cf.ac.uk/ac/index.php). All the variants have been located in two intracellular kinase domains (exons 17–26) [3, 31, 32] and are presumed to interfere with tyrosine kinase activation [6, 7, 25–30] of the *FLT4/VEGFR3* receptor, which impairs the VEGF-C/D-VEGFR3 pathway. This results in f the cutaneous lymphatic network hypoplasia [33] and finally leads to MD phenotypes. In this study, we sequenced the tyrosine kinase coding domains of *FLT4/VEGFR3* in a large Chinese family with hereditary congenital lymphoedema and found a missense variant that led to a valine-to-glutamic acid substitution (Fig. 4). As previously noted, mutant FLT4/VEGFR3 proteins are poor activators of downstream signalling cascades because the missense variant has no tyrosine activity. The wild-type receptor is internalized and degraded at a faster rate and maintained for a shorter time at the cell surface than the mutant type[6]. Therefore, the amount of the mutant receptor on the endothelial cell surface would be considerably higher and may contribute to the development of lymphoedema by reducing the relative amount of ligand binding to the active wild-type *FLT4/VEGFR3*. Such a “dominant-negative” effect might lead to hypoplastic and dysfunctional cutaneous lymphatic vessels [6, 12], which fail to transport fluid into the venous circulation, resulting in lymphatic fluid stasis and swelling of the extremities [34–37]. The increase in interstitial protein-rich fluid leads to insufficient lymphatic drainage and transport [38], causes a large amount of protein-rich fluid to accumulate in the tissue interstitial spaces and the hyperplasia of the skin, subcutaneous tissue, and fibrous tissues, and finally causes lymphatic accumulation, making it difficult to reflux to lymphatic vessels. Meanwhile, oedematous fluid and adipose tissue accumulate subcutaneously, followed by an inflammatory response that develops and forms a vicious cycle that aggravates the formation of oedema [39, 40]. Moreover, retarded lymphatic flow-induced lipogenesis and fat deposition lead to increased fibrocyte and connective tissue overgrowth [41–43]. Then, the affected skin thickens, hardens, and becomes rough and bulky, forming “elephant skin” over time.

Researchers found that variants affecting conserved residues in the tyrosine kinase domain (residues 843–943 and 1,009–1,165) are correlated with a severe form of lymphoedema[44]. However, in our MD family, the phenotypes were quite different among patients with the same variant. Only two patients in this family suffered from relatively severe clinical phenotypes compared with the other members. Patient I1 complained that oedema of both legs was aggravated and extended to the roots of the thighs when he had a cold and fever in childhood, and patient II1 suffered from heavy oedema of the lower extremities and an “elephant-like” skin hyperkeratosis of her legs (Fig. 2-1, 2) [4, 45, 46]. In contrast, neither the brawny texture of the skin nor lymphoedema was difficult to observe among some patients (II2, II4 and III4). How this novel missense variant impaired the tyrosine kinase and VEGF-C/D/VEGFR3 signalling pathways is not totally clear, nor are the potential reasons for the phenotypic heterogeneities in our MD family. Further research is needed to explore the causative factors of the heterogeneities. Genetic variants might not be the only factor shaping the clinical phenotypes of MD, and environmental, genetic, and epigenetic factors and their interactions should be considered.

## Conclusion

In this report, we described a large Chinese family with manifestations of MD caused by a missense variant in *FLT4/VEGFR3* (NM_182925.4 (*FLT4/VEGFR3*_v001):c.2774 T>A, p. (Val925Glu)). There are phenotypic heterogeneities among family members, and further research should be conducted to explore the possible reasons.

## Supplementary Information

**Additional file 1: Figure S1.** Hph I restriction enzyme digestion results of FLT4 gene. Legend of Figure S1-1, 2, 3: Digests of FLT4 gene amplicons from family members. In this pedigree, the mutation creates an HphI restriction site. Digests of FLT4 amplicon (262 bp) from affected individuals fractionate into three fragments (262 bp, 198 bp and 64 bp). FLT4 amplicon (262 bp) from healthy individuals showed only one fragment: the 262 bp fragment. M indicates DNA marker. NC: Normal control

**Additional file 2: Figure S2.** Conserved motif of FLT4 Protein. Legend of Figure S2: Amino acid alignment around the affected residue of the FLT4 protein. The highly conserved V925 is marked by black arrow

**Additional file 3: Figure S3.** Polygen Tree of FLT4 gene. Legend of Figure S3: The invariant 925 valine in the tyrosine kinase domain and the wild type is conserved in a wide range of organisms, ranging from humans to Danio_rerio

**Additional file 4: Table S1.** PCR amplification system and conditions of tyrosine kinase coding domains of FLT4 gene

**Additional file 5: Table S2.** The pathogenic prediction results of silico program

**Additional file 6: Table S3.** Review of clinical phenotypes of Milroy disease

## Data Availability

The raw datasets used and analysed during the current study are not deposited in publicly available repositories because of considerations about the security of human genetic resources. The transcript RefSeq number was obtained from the NCBI database (https://www.ncbi.nlm.nih.gov/gene/). For other details of the availability of data and material, please refer to the methods section of the article and Additional files. Any questions should be directed to the corresponding author.

## Abbreviations

MD: Milroy disease
FLT4/VEGFR3: Fms-related tyrosine kinase 4/ vascular endothelial growth factor receptor 3
PCR: Polymerase chain reaction
PCL: Primary congenital lymphedema
