# Supplementary figures and images for: A family with Milroy disease caused by the FLT4/VEGFR3 gene variant c.2774 T > A
Source: BMC Med Genomics. 2021 Jun 8;14:151. doi: 10.1186/s12920-021-00997-w (PMC8186030; doi:10.1186/s12920-021-00997-w)

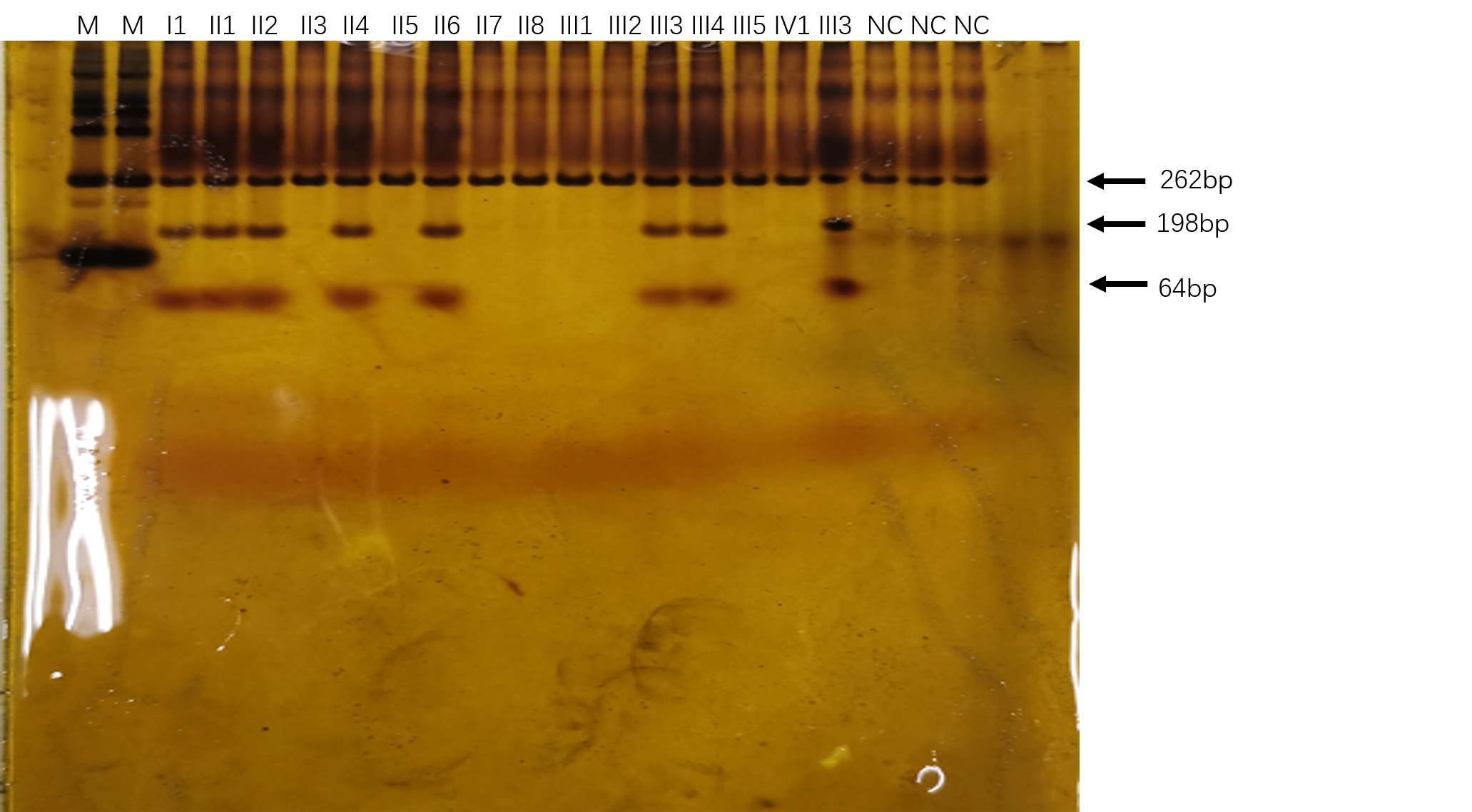

Supplement: Supplementary file 1 — Additional file 1: Figure S1. Hph I restriction enzyme digestion results of FLT4 gene. Legend of Figure S1-1, 2, 3: Digests of FLT4 gene amplicons from family members. In this pedigree, the mutation creates an HphI restriction site. Digests of FLT4 amplicon (262 bp) from affected individuals fractionate into three fragments (262 bp, 198 bp and 64 bp). FLT4 amplicon (262 bp) from healthy individuals showed only one fragment: the 262 bp fragment. M indicates DNA marker. NC: Normal control [file 12920_2021_997_MOESM1_ESM.tif]

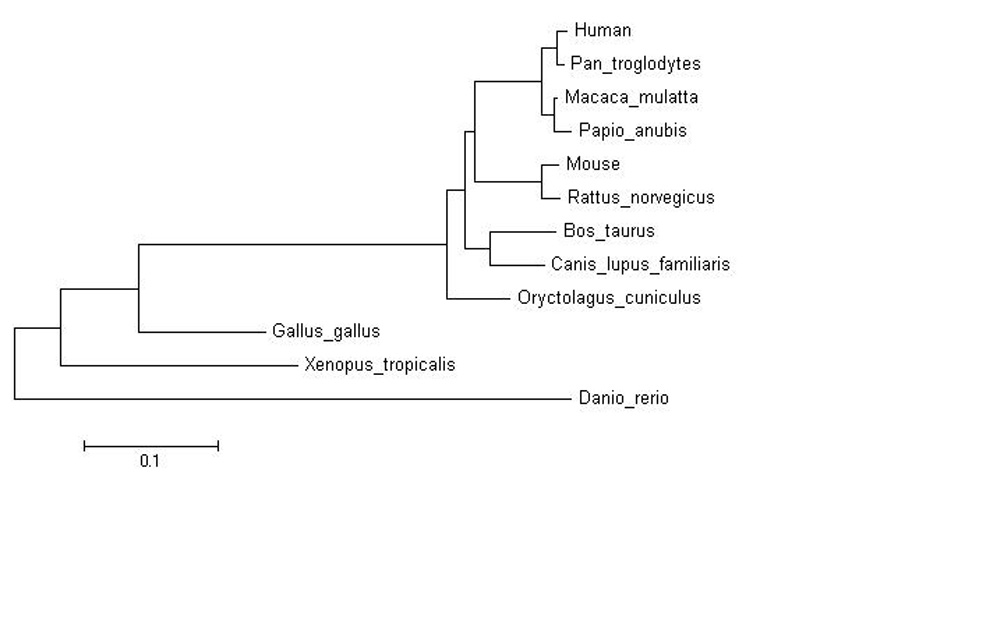

Supplement: Supplementary file 2 — Additional file 2: Figure S2. Conserved motif of FLT4 Protein. Legend of Figure S2: Amino acid alignment around the affected residue of the FLT4 protein. The highly conserved V925 is marked by black arrow [file 12920_2021_997_MOESM2_ESM.jpg]

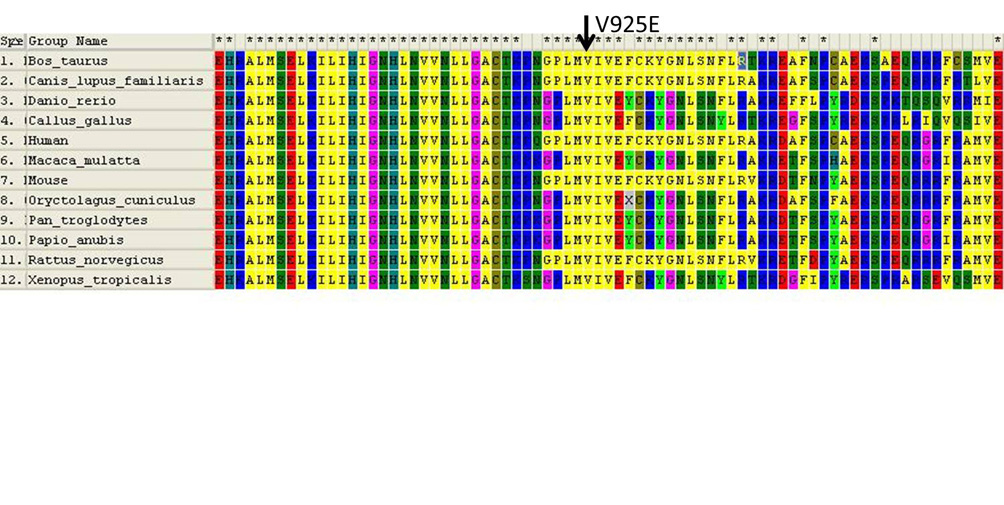

Supplement: Supplementary file 3 — Additional file 3: Figure S3. Polygen Tree of FLT4 gene. Legend of Figure S3: The invariant 925 valine in the tyrosine kinase domain and the wild type is conserved in a wide range of organisms, ranging from humans to Danio_rerio [file 12920_2021_997_MOESM3_ESM.jpg]
