# Supplementary material for: A family with Milroy disease caused by the FLT4/VEGFR3 gene variant c.2774 T > A
Source: BMC Med Genomics. 2021 Jun 8;14:151. doi: 10.1186/s12920-021-00997-w (PMC8186030; doi:10.1186/s12920-021-00997-w)
Supplement: Supplementary file 4 — Additional file 4: Table S1. PCR amplification system and conditions of tyrosine kinase coding domains of FLT4 gene [file 12920_2021_997_MOESM4_ESM.doc]

| Table S1. PCR amplification system and conditions of tyrosine kinase coding domains of *FLT4/VEGFR3* gene | | | | | | |
| --- | --- | --- | --- | --- | --- | --- |
| Exons | Forward Primer (5’-3’) | Reverse Primer (5’-3’) | Annealing temperature(℃) | Product length (bp) | PCR amplification system | PCR amplification condition |
| Exon 17 | TGGCCGCCCCTAAGATAAAG | ATTCGCATTGCTCCTCCAGA | 58.0 | 330 | Denaturing: 95℃ for 5 min;  35 cycles :  Denaturing at 95℃ 30s  Annealing at 58℃ 30s  Extension at 72℃ 30s  Final step at 72℃ 7 min | 2×Biotech Power PCR Mix: 10µl;  forward primer: 0.8µl (10µM);  reverse primer: 0.8µl (10µM);  DNA template: 1µl (50ng/µl);  ddH20: 12.4µl. |
| Exon 18-19 | CTCTCCTTGTCTTCCCGCTG | GGCCGTGCGTTCGGA | 58.0 | 788 | Denaturing: 95℃ for 5 min;  35 cycles :  Denaturing at 95℃ 30s  Annealing at 58℃ 30s  Extension at 72℃ 30s  Final step at 72℃ 7 min | 2×LA Taq GC buffer II 8.0μl  4×dNTP 2.0μl  LA Taq 0.5μl  DNA 1.0μl  Forward Primer 0.5μl  Reverse Primer 0.5μl  ddH_2_O: 12.5μl |
| Exon 20 | CTTCATCAGCGTCGAGTGG | ATTATGGGCGGGTTCCTT | 58.0 | 176 | Denaturing: 95℃ for 5 min;  35 cycles :  Denaturing at 95℃ 30s  Annealing at 58℃ 30s  Extension at 72℃ 30s  Final step at 72℃ 7 min | 2×Biotech Power PCR Mix: 10µl;  forward primer: 0.8µl (10µM);  reverse primer: 0.8µl (10µM);  DNA template: 1µl (50ng/µl);  ddH20: 12.4µl. |
| Exon21 | TGCAAGTACGGCAACCTCTC | GCCTCTGATGACCCAGTCAA | 60.0 | 508 | Denaturing: 95℃ for 5 min;  35 cycles :  Denaturing at 95℃ 30s  Annealing at 60℃ 30s  Extension at 72℃ 30s  Final step at 72℃ 7 min | 2×Biotech Power PCR Mix: 10µl;  forward primer: 0.8µl (10µM);  reverse primer: 0.8µl (10µM);  DNA template: 1µl (50ng/µl);  ddH20: 12.4µl. |
|  |  |  |  |  |  |  |

Table S1. PCR amplification system and conditions of tyrosine kinase coding domains of *FLT4/VEGFR3* gene

| Exons | Forward Primer (5’-3’) | Reverse Primer (5’-3’) | Annealing temperature  (℃) | | Product length (bp) | | PCR amplification system | PCR amplification condition | |
| --- | --- | --- | --- | --- | --- | --- | --- | --- | --- |
| Exon 22 | GGAGAGGGGCTGACATGC | AACAGACTCTGCTGCTGACC | 53.0 | | 392 | | Denaturing: 95℃ for 5 min;  35 cycles :  Denaturing at 95℃ 30s  Annealing at 53℃ 30s  Extension at 72℃ 30s  Final step at 72℃ 7 min | 2×Biotech Power PCR Mix: 10µl;  forward primer: 0.8µl (10µM);  reverse primer: 0.8µl (10µM);  DNA template: 1µl (50ng/µl);  ddH20: 12.4µl. | |
| Exon 23 | CAAAGGAGCACAGTTCAGGC | CACTTCCTTGCCCCAAGATG | 58.0 | | 460 | | Denaturing: 95℃ for 5 min;  35 cycles :  Denaturing at 95℃ 30s  Annealing at 58℃ 30s  Extension at 72℃ 30s  Final step at 72℃ 7 min | 2×Biotech Power PCR Mix: 10µl;  forward primer: 0.8µl (10µM);  reverse primer: 0.8µl (10µM);  DNA template: 1µl (50ng/µl);  ddH20: 12.4µl. | |
| Exon  24 | ACAGTCTACCCCGATACCCC | TTCAATGTGCACCCCACAGA | 55.0 | | 630 | | Denaturing: 95℃ for 5 min;  35 cycles :  Denaturing at 95℃ 30s  Annealing at 55℃ 30s  Extension at 72℃ 30s  Final step at 72℃ 7 min | 2×Biotech Power PCR Mix: 10µl;  forward primer: 0.8µl (10µM);  reverse primer: 0.8µl (10µM);  DNA template: 1µl (50ng/µl);  ddH20: 12.4µl. | |
| Exon  25 | GTAGAGCACAGTCGGGTGAG | TTTCCCTGAACACTCGGCTC | 55.0 | | 477 | | Denaturing: 95℃ for 5 min;  35 cycles :  Denaturing at 95℃ 30s  Annealing at 55℃ 30s  Extension at 72℃ 30s  Final step at 72℃ 7 min | 2×Biotech Power PCR Mix: 10µl;  forward primer: 0.8µl (10µM);  reverse primer: 0.8µl (10µM);  DNA template: 1µl (50ng/µl);  ddH20: 12.4µl. | |
| Table S1. PCR amplification system and conditions of tyrosine kinase coding domains of *FLT4//VEGFR3* gene | | | | | | | | | |
| Exons | Forward Primer (5’-3’) | Reverse Primer (5’-3’) | | Annealing temperature  (℃) | | Product length (bp) | PCR amplification system | PCR amplification condition | |
| Exon  26 | CCACCCTCAAGATACACGGG | AGGTGGGCAGAGCCTAGAT | | 55.0 | | 489 | Denaturing: 95℃ for 5 min;  35 cycles :  Denaturing at 95℃ 30s  Annealing at 55℃ 30s  Extension at 72℃ 30s  Final step at 72℃ 7 min | 2×Biotech Power PCR Mix: 10µl;  forward primer: 0.8µl (10µM);  reverse primer: 0.8µl (10µM);  DNA template: 1µl (50ng/µl);  ddH20: 12.4µl. | |
| Exons  27 | GTGCTGGTTTCTGGCATGAG | GACCCTGCAGTGGGTCATTTA | | 60.0 | | 589 | Denaturing: 95℃ for 5 min;  35 cycles :  Denaturing at 95℃ 30s  Annealing at 60℃ 30s  Extension at 72℃ 30s  Final step at 72℃ 7 min | 2×Biotech Power PCR Mix: 10µl;  forward primer: 0.8µl (10µM);  reverse primer: 0.8µl (10µM);  DNA template: 1µl (50ng/µl);  ddH20: 12.4µl. | |
|  | | | | | | | | |  |
