# Supplementary material for: A family with Milroy disease caused by the FLT4/VEGFR3 gene variant c.2774 T > A
Source: BMC Med Genomics. 2021 Jun 8;14:151. doi: 10.1186/s12920-021-00997-w (PMC8186030; doi:10.1186/s12920-021-00997-w)
Supplement: Supplementary file 5 — Additional file 5: Table S2. The pathogenic prediction results of silico program [file 12920_2021_997_MOESM5_ESM.doc]

Table S2. The pathogenic prediction results of silico program

| In silico program | Website | Results | Explanation |
| --- | --- | --- | --- |
| SIFT | http://sift.jcvi.org | 0.00 | deleterious |
| PolyPhen | http://genetics.bwh.harvard.edu/pph2/ | 1.00 | damaging |
| Mutaiontaster | http://www.mutationtaster.org/ | Disease  causing | pathogenic |
| MutaionAssessor | http://www.mutationassessor.org/r3 | media | predicted functional |
| fathmm | http://fathmm.biocompute.org.uk/ | Damaging | damaging |
| PROVEN | http://provean.jcvi.org/seq_submit.php | -5.084 | Deleterious |
| M-CAP | <http://bejerano.stanford.edu/MCAP/index.html> | 0.937 | Possibly Pathogenic |
