# Supplementary material for: A family with Milroy disease caused by the FLT4/VEGFR3 gene variant c.2774 T > A
Source: BMC Med Genomics. 2021 Jun 8;14:151. doi: 10.1186/s12920-021-00997-w (PMC8186030; doi:10.1186/s12920-021-00997-w)
Supplement: Supplementary file 6 — Additional file 6: Table S3. Review of clinical phenotypes of Milroy disease [file 12920_2021_997_MOESM6_ESM.doc]

Table S3. Review of clinical phenotypes of Milroy disease

| Year, author | Clinical phenotypes |
| --- | --- |
| 2000, Irrthum et al. | bilateral lymphedema of lower limb  erysipelas  prominent veins  hyperbilirubinism  epicanthnic folds |
| 2005,Brice et al. | Lymphedema present : 90%  Congenital lymphedema: 97%  Non-congenital: 3%  Blow knee edema: 94%  Above knee edema: 6%  Bilateral edema: 85%  Unilateral edema: 15%  Hydrocoele: 37%  Prominent veins: 23%  “ski jump” toenails: 14%  Papillomatosis: 10% |
| 2012, Gezginc et al. | Bilateral leg edema |
|  | hydrocele,  hyperkeratosis,  ski jump toenails  papillomatosis.  preputial oedema |
|  | lymphedema of both feet and legs, involved dorsum of feet |
| 2014, DiGiovanni et al. | bilateral, below the knee swelling |
| 2015,Melikhan-Revzin et al. | Lower extremities edema  Testes edema |
|  | |

Table S2. Review of clinical phenotypes of Milroy disease

| Year, author | Clinical phenotypes |
| --- | --- |
| 2005,Mizuno | bilateral lower limb swelling limited to dorsum of the foot  bilateral swelling of foot |
|  | Case 1  fetal edema of the lower limbs and ascites  Bilateral pleural effusion  major edema of the lower limbs, hands and genitalia at birth  discrete lymphedema of the dorsum of both hands and fingers.  Case 2  lymphedema of both feet and legs and hydrocele  large caliber leg |
| 2007,Carver | Patient 1 presented at 21 months.  lower limb to the ankle but predominantly the dorsum of the feet and the toes  small dysplastic upturned toenails, deep creases, and swollen sausage like toes.  Large calibre vein on the dorsum of the right foot.  Patient 2 was seen at the age of 21 months.  Swelling of his left leg was noted below the knee from birth. Swelling of the right lower leg was noted later.  widespread eczema, particularly over the feet  Patient 3 was seen at the age of 5.5 years  born with swelling in both feet.  Infected toenails and recurrent cellulitis. |
| 2009, Ghalamkarpour  et al. | Patient 1  pleural effusion  chylous  ascites, or polyhydramnios.  bilateral lower limb lymphedema.  Patient 2  edema of lower limbs.  Patient 3  a cystic hygroma at 18 weeks’ gestation.  At 30 weeks there was generalized subcutaneous lymphedema, massive pleural effusion, minor ascites, and polyhydramnios.  After birth, the patient had left ptosis, distichiasis, low posterior hairline, webbed necked and short left second toe.  Systemic lymphedema resolved over 10 days and chylothorax resorbed  by 4 months of age. No other abnormalities were detected. At 9 years of age, the patient did not have lymphedema. |

Table S2. Review of clinical phenotypes of Milroy disease

| Year, author | Clinical phenotypes | |
| --- | --- | --- |
| 2008, Futatani | Family 1  bilateral lower limb swelling limited to dorsum of the foot since birth.  Family 2  lower limb swelling of the bilateral foot | |
| References: | |  |

1. Irrthum, A., et al., *Congenital hereditary lymphedema caused by a mutation that inactivates VEGFR3 tyrosine kinase.* Am J Hum Genet, 2000. **67**(2): p. 295-301.

2. Brice, G., et al., *Milroy disease and the VEGFR-3 mutation phenotype.* J Med Genet, 2005. **42**(2): p. 98-102.

3. Gezginc, K., F. Yazici, and D. Gok, *Prenatal diagnosis of Milroy's primary congenital lymphedema.* Taiwan J Obstet Gynecol, 2012. **51**(2): p. 280-2.

4. DiGiovanni, R.M., et al., *A novel FLT4 mutation identified in a patient with Milroy disease.* Lymphology, 2014. **47**(1): p. 44-7.

5. Melikhan-Revzin, S., et al., *A Novel Missense Mutation in FLT4 Causes Autosomal Recessive Hereditary Lymphedema.* Lymphat Res Biol, 2015. **13**(2): p. 107-11.

6. Mizuno, S., et al., *Clinical variability in a Japanese hereditary lymphedema type I family with an FLT4 mutation.* Congenit Anom (Kyoto), 2005. **45**(2): p. 59-61.

7. Carver, C., et al., *Three children with Milroy disease and de novo mutations in VEGFR3.* Clin Genet, 2007. **71**(2): p. 187-9.

8. Ghalamkarpour, A., et al., *Recessive primary congenital lymphoedema caused by a VEGFR3 mutation.* J Med Genet, 2009. **46**(6): p. 399-404.

9. Futatani, T., et al., *Molecular characterization of two novel VEGFR3 mutations in Japanese families with Milroy's disease.* Pediatr Int, 2008. **50**(1): p. 116-8.
